# Pre- and posttreatment with hydrogen sulfide prevents ventilator-induced lung injury by limiting inflammation and oxidation

**DOI:** 10.1371/journal.pone.0176649

**Published:** 2017-04-28

**Authors:** Simone Faller, Raphael Seiler, Rosa Donus, Helen Engelstaedter, Alexander Hoetzel, Sashko Gregoriev Spassov

**Affiliations:** Department of Anesthesiology and Critical Care Medicine, University Medical Center Freiburg, Freiburg, Germany; University of Louisville, UNITED STATES

## Abstract

Although essential in critical care medicine, mechanical ventilation often results in ventilator-induced lung injury. Low concentrations of hydrogen sulfide have been proven to have anti-inflammatory and anti-oxidative effects in the lung. The aim of this study was to analyze the kinetic effects of pre- and posttreatment with hydrogen sulfide in order to prevent lung injury as well as inflammatory and oxidative stress upon mechanical ventilation. Mice were either non-ventilated or mechanically ventilated with a tidal volume of 12 ml/kg for 6 h. Pretreated mice inhaled hydrogen sulfide in low dose for 1, 3, or 5 h prior to mechanical ventilation. Posttreated mice were ventilated with air followed by ventilation with hydrogen sulfide in various combinations. In addition, mice were ventilated with air for 10 h, or with air for 5 h and subsequently with hydrogen sulfide for 5 h. Histology, interleukin-1β, neutrophil counts, and reactive oxygen species formation were examined in the lungs. Both pre-and posttreatment with hydrogen sulfide time-dependently reduced or even prevented edema formation, gross histological damage, neutrophil influx and reactive oxygen species production in the lung. These results were also observed in posttreatment, when the experimental time was extended and hydrogen sulfide administration started as late as after 5 h air ventilation. In conclusion, hydrogen sulfide exerts lung protection even when its application is limited to a short or delayed period. The observed lung protection is mediated by inhibition of inflammatory and oxidative signaling.

## Introduction

Despite advances in ventilator therapy over the last two decades [1], the risk that mechanical ventilation will induce or aggravate lung injury (i.e., ventilator-induced lung injury (VILI)) remains unacceptably high [2]. This is not only true for ventilated patients with pre-existing lung disease, but VILI also occurs during ventilation of healthy lungs [3,4]. VILI is characterized by lung tissue disruption, edema formation, excessive production of reactive oxygen species (ROS), and the development of an inflammatory response. Two mechanisms appear to play key roles within the process of VILI: first, constant cyclic stretching of the lungs can release pro-inflammatory cytokines such as interleukin-1β (IL-1β), and trigger the transmigration of neutrophil cells into the alveolar compartments [5,6]. Second, the excessive production of ROS upon mechanical ventilation may per se damage lung tissue by peroxidation of cell lipids, DNA breakage, or alteration of amino acids and cellular metabolism [7,8]. In order to limit both of these responses to mechanical ventilation and thus reduce lung injury, a treatment option targeted at these mechanisms would be highly desirable.

In this respect, hydrogen sulfide (H_2_S) is of emerging interest. Together with nitric oxide and carbon monoxide, H_2_S belongs to the family of so called endogenous gasotransmitters [9]. Given exogenously, its organ- and also lung-protective potential has been proven in various experimental models in the past [10–17]. We and others have previously shown that H_2_S inhalation prevents acute lung injury in a model of pulmonary inflammation [12], hyperoxic lung injury [13], ischemia-reperfusion injury [15], or ventilator-induced lung injury [10,11,16,17]. In these models, low concentrations of H_2_S limited inflammatory responses by preventing the release of pro-inflammatory cytokines and neutrophil accumulation in the lung. Moreover, oxidative stress was found to be clearly decreased in response to H_2_S application [13–15,17,18].

In earlier studies lung protection as well as anti-inflammatory or anti-oxidative effects were observed when H_2_S administration continued over the entire experimental course [10,11,16,17]. Up to now, only in ischemia-reperfusion models, pre- or postconditioning with H_2_S have been described, resulting in protection from cardiac (reviewed in [19]), retinal [20], intestinal [21–23], hepatic [24], or neuronal insults [25]. However, virtually nothing is known about time-dependent effects of H_2_S treatment in lung injury.

Here, we determined the time-dependent anti-inflammatory and anti-oxidative effects of inhaled H_2_S as pre- and posttreatment in a well-established mouse VILI-model. Our results provide evidence that H_2_S can protect mice from VILI by limiting inflammatory and oxidative responses when given prior to the onset of mechanical ventilation and also when the onset of H_2_S application is delayed during the course of injurious ventilation.

## Materials and methods

### Animals

Animal experiments were performed in accordance with the guidelines of the local animal care commission (University of Freiburg, Freiburg, Germany) and in conformance with the journals’ requirements for human and animal trials (ARRIVE guidelines). The study was approved by the local government, which had been advised by an ethics committee (Regierungspräsidium Freiburg, Freiburg, Germany, permission No. G-12/73). All surgery was performed under deep ketamine and acepromazine anesthesia, and all efforts were made to minimize suffering.

### Experimental setup

Male C57BL/6N mice were obtained from Charles River Laboratories (Sulzfeld, Germany). Mice were anesthetized intraperitoneally (i.p., 90 mg/kg ketamine and 0.9 mg/kg acepromazine). As described earlier [26], an arterial line and a tracheal tube were inserted. In the case of mechanical ventilation, mice were connected to a rodent ventilator (Voltekenterprises, Toronto, ON, Canada), set as follows: tidal volume of 12 ml/kg, frequency of 80–90 breaths/minute, positive end-expiratory pressure of 2 cmH_2_O for 6 hours. Alveolar recruitment maneuvers were performed every 60 minutes (5 seconds of inspiratory pressure hold at 30 cmH_2_O). Directly after the onset of mechanical ventilation, muscular relaxation was induced by injection of pancuronium (2 mg/kg, i.p.) and maintained during the experiment by injection as needed. Likewise, general anesthesia was maintained by continuous i.p. administration of ketamine and acepromazine as needed. Peak pressure (Ppeak), plateau pressure (Pplateau), and mean arterial pressure (MAP) were monitored throughout the experiment; blood gas analyses were performed at the beginning (after 30 min of ventilation) and the end of the ventilation period (6 h). At the end of each experiment, mice were sacrificed by an overdosed injection of ketamine and acepromazine. Bronchoalveolar lavage (BAL) fluid was gained by flushing the right lung lobes. The lungs were preserved for histological examination as described previously [26].

**Fig 1:**
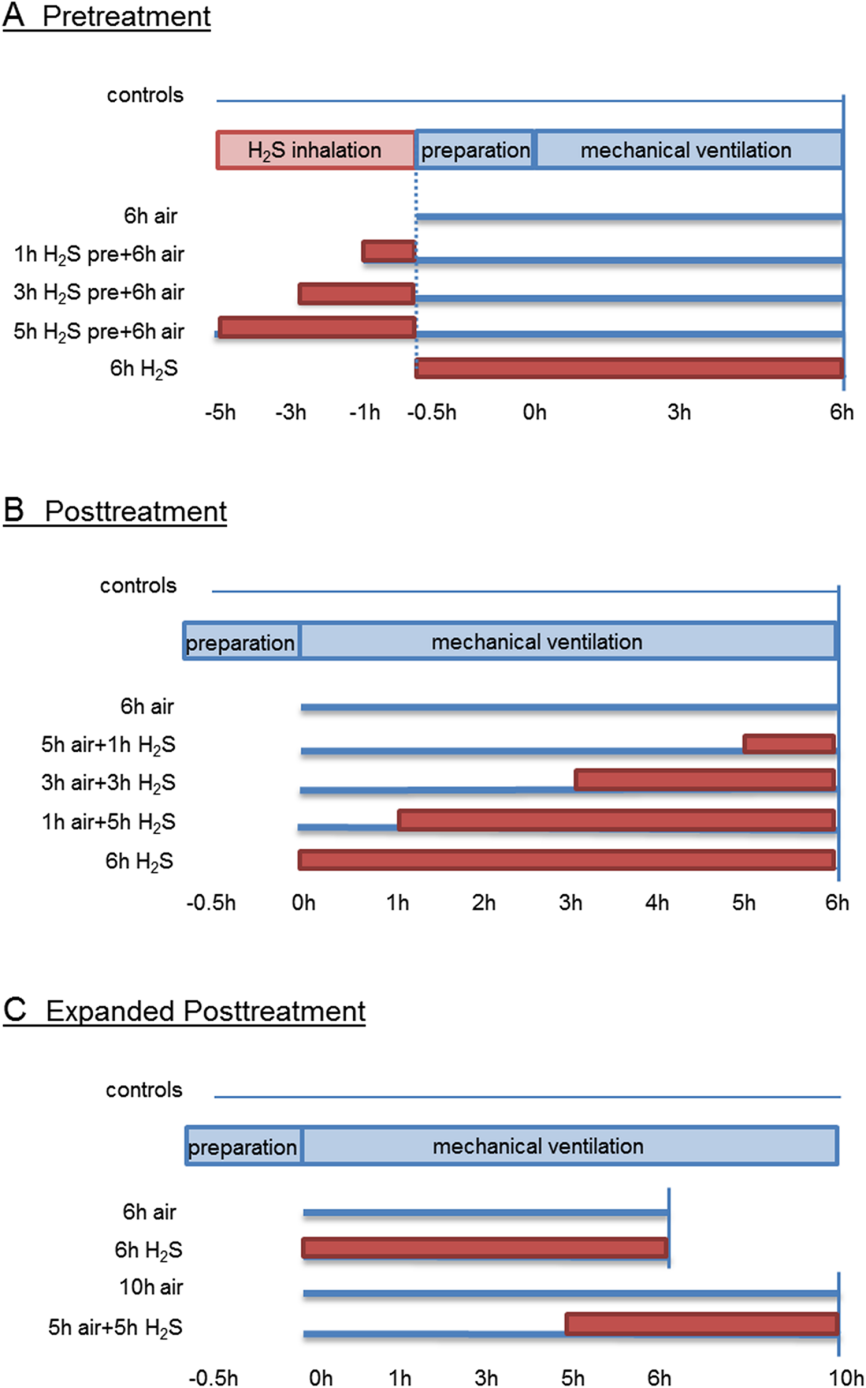
Study design and timeline. (A) **Pretreatment.** Mice spontaneously breathed air (control) for 6 h or were mechanically ventilated with 12 ml/kg for 6 h with either air alone (6 h air) or air supplemented with 80 ppm H_2_S (6 h H_2_S). All other mice spontaneously breathed air supplemented with 80 ppm H_2_S for 1, 3, or 5 h prior to mechanical ventilation with air for another 6 h as indicated. (B) **Posttreatment.** Mice spontaneously breathed air (control) for 6 h or were mechanically ventilated with 12 ml/kg for 6 h with either air alone (6 h air) or air supplemented with 80 ppm H_2_S (6 h H_2_S). All other mice were first mechanically ventilated with air alone for 5, 3, or 1 h, followed by ventilation with 80 ppm H_2_S for another 1, 3, or 5 h as indicated. (C) **Expanded Posttreatment**. Mice spontaneously breathed air (control) for 6 h or were mechanically ventilated with 12 ml/kg with either air in the absence or presence of 80 ppm H_2_S (6 h air, 6 h H_2_S) or were ventilated for 10 h with air alone (10 h air) or for 5 h with air alone followed by ventilation with H_2_S for another 5 h (5 h air + 5 h H_2_S).

The animal experiments were run in three independent experimental sets (Fig 1):

#### Set 1 Pretreatment (n = 7/group; 6 groups)

The control group spontaneously breathed air (control) for 6 hours. All other mice were either ventilated with synthetic air (6h air) or subjected to spontaneously breathe 80 parts per million (ppm) H_2_S for 1, 3, or 5 h prior to another 6 h ventilation with synthetic air (1 h H_2_S pre + 6 h air; 3 h H_2_S pre + 6 h air; 5 h H_2_S pre + 6 h air) or they were ventilated with synthetic air supplemented with 80 ppm H_2_S (6 h H_2_S; Fig 1A).

#### Set 2 Posttreatment (n = 7/group; 6 groups)

The control group spontaneously breathed air (control) for 6 hours. All other mice were either ventilated with synthetic air (6 h air) or they were first ventilated with synthetic air for 5, 3, or 1 h and then 80 ppm H_2_S was supplemented for another 1, 3, or 5 h (5 h air + 1 h H_2_S; 3 h air + 3 h H_2_S; 1 h air + 5 h H_2_S) or they were ventilated with synthetic air supplemented with 80 ppm H_2_S (6 h H_2_S; Fig 1B).

#### Set 3 expanded posttreatment (n = 6/group; 5 groups)

The control group spontaneously breathed air (control) for 6 hours. All other mice were either ventilated with synthetic air (6 h air), synthetic air supplemented with 80 ppm H_2_S (6 h H_2_S), or with synthetic air for 10 h (10 h air), or they were first ventilated with synthetic air for 5 h and then 80 ppm H_2_S was supplemented for another 5 h (5 h air + 5 h H_2_S; Fig 1C).

### Histological analysis

Cryosections of the fixed left lung were stained with hematoxylin and eosin (H&E) and analyzed for alveolar wall thickness and VILI score as previously described [26].

### Neutrophil and cytokine analysis

BAL fluid cells were separated and analyzed as described previously [11]. BAL supernatant was analyzed using an interleukin-1β (IL-1β) ELISA kit (R&D Systems GmbH, Wiesbaden, Germany) according to the manufacturers’ instructions.

### Detection of reactive oxygen species (ROS)

Cryosections of unfixed frozen lung tissue samples were stained with dihydroethidium (Life Technologies GmbH, Darmstadt, Germany) and analyzed as described previously in order to detect ROS [13,17,26].

### Statistical analysis

All animal experiments were performed with n = 7 (Set 1 and 2) or n = 6 (Set 3) mice per group as indicated. Power calculations were performed prior to the study in order to define group sizes. Graphs represent means ± standard error of means (SEM) and were created with SigmaPlot 11.0 (Systat Software Inc., Erkrath, Germany). Data were further analyzed for normal variation prior to one way analysis of variance (ANOVA) followed by the Tukey`s post hoc test. P<0.05 was considered significant. All calculations were performed with GraphPad Prism 5 (GraphPad Software, Inc., La Jolla, CA USA).

## Results

### Effect of H_2_S pretreatment on physiological parameters

Blood gas analysis for Pa_O2_, Pa_CO2,_ and pH at the end of the ventilation period did not differ between 6 h air, 6 h H_2_S ventilated, or 1, 3, 5 h H_2_S pretreated groups, followed by additional 6 h air ventilation (S1 Table). With respect to circulatory function, only after 5 h of H_2_S preconditioning followed by 6 h air ventilation, mean arterial pressure was reduced compared to 6 h air ventilation alone, while mean arterial pressure (MAP) in all other groups did not vary. Peak pressure, plateau pressure, and static compliance did not differ between groups (S1 Table).

### Effect of H_2_S pretreatment on ventilator-induced lung injury

**Fig 2:**
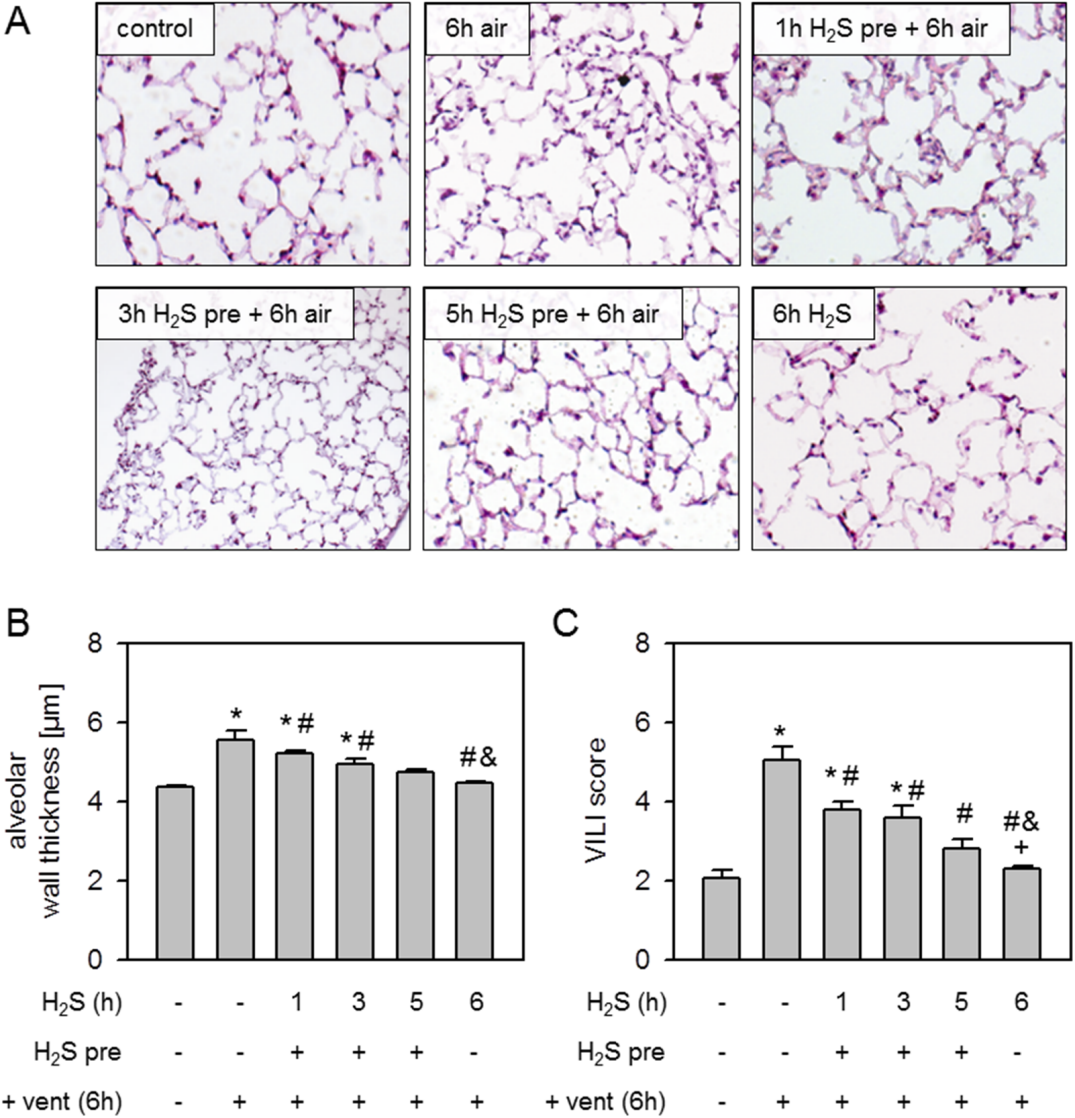
Effect of H_2_S pretreatment on ventilator-induced lung injury. Mice spontaneously breathed air (control) or for 6 h, or they were mechanically ventilated with 12 ml/kg for 6 h either with air alone (6 h air) or air supplemented with 80 ppm H_2_S (6 h H_2_S). All other mice spontaneously breathed air supplemented with 80 ppm H_2_S 1, 3, or 5 h prior to mechanical ventilation with air for another 6 h as indicated. Lung sections were stained with H&E. Representative pictures are shown for each experimental group as indicated (A). Alveolar wall thickness was measured (B) and ventilator-induced lung injury (VILI) score was calculated (C). Data represent means ± SEM for n = 7/group. ANOVA (Tukey`s post hoc test), *P<0.05 vs. control group; ^#^*P*<0.05 vs. 6h air vent group; ^&^*P*<0.05 vs. 1h H_2_S pre + 6h air vent group.

Compared to non-ventilated controls, mechanical ventilation with air for 6 h caused alveolar wall thickening, i.e., edema formation (Fig 2A). Pretreatment with inhalative H_2_S for 1 h prior to mechanical ventilation only marginally reduced alveolar wall thickening, while pretreatment with H_2_S for 3 or 5 h diminished alveolar wall thickening back to control levels (Fig 2A). Likewise, 6 h ventilation with H_2_S completely prevented the development of alveolar wall thickening, which was also confirmed by quantitative analysis (Fig 2B). Similar results were obtained by calculating an overall VILI score (Fig 2C). While mechanical ventilation with air alone led to a significantly augmented VILI score compared to controls and all other treatments, there was a stepwise reduction from 1 and 3 h H_2_S pretreatment to 5 h H_2_S pretreatment and 6 h H_2_S ventilation. The VILI scores of the latter two groups were comparable to control levels (Fig 2C).

### Effect of H_2_S pretreatment on lung inflammation and oxidative stress

**Fig 3:**
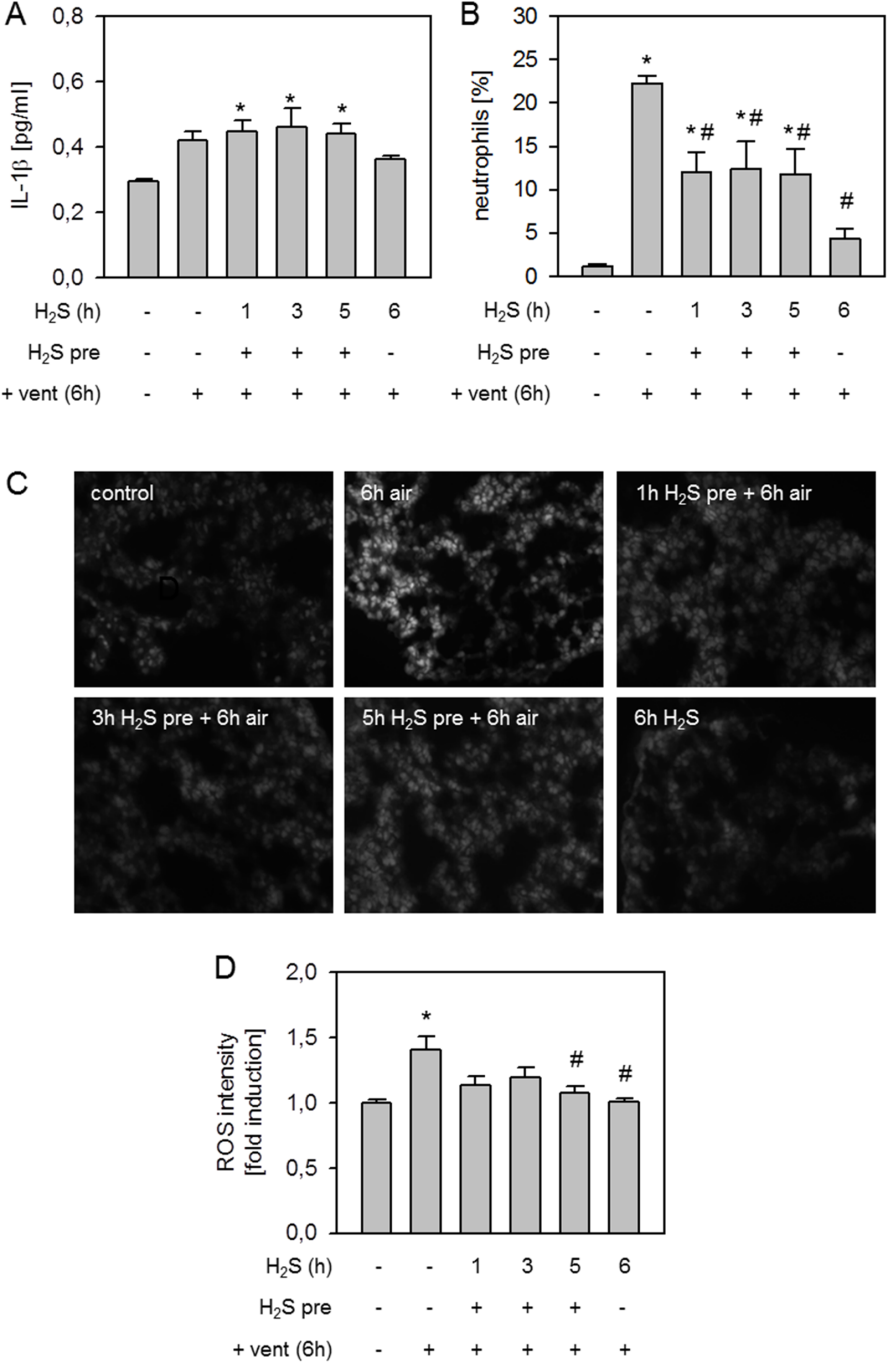
Effect of H_2_S pretreatment on lung inflammation and oxidative stress. Mice spontaneously breathed air (control) or for 6 h, or they were mechanically ventilated with 12 ml/kg for 6 h either with air alone (6 h air) or air supplemented with 80 ppm H_2_S (6 h H_2_S). All other mice spontaneously breathed air supplemented with 80 ppm H_2_S 1, 3, or 5 h prior to mechanical ventilation with air for another 6 h as indicated. Bronchoalveolar lavage (BAL) IL-1β cytokine content was determined by ELISA (A). The fraction of neutrophil cells was measured in BAL fluid by cytospin analysis (B). Lung tissue sections were stained with dihydroethidium (C). Representative pictures are shown for each experimental group as indicated in C. ROS fluorescence intensity was measured, calculated, and expressed as fold induction compared to control group (D). Data represent means ± SEM for n = 6/group. ANOVA (Tukey`s post hoc test), *P<0.05 vs. control group; ^#^*P*<0.05 vs. 6h air vent group.

With respect to lung inflammatory parameters, we observed a slight increase in the release of the pro-inflammatory cytokine IL-1β following 6 h air ventilation and in all H_2_S pretreated groups compared to controls. In the 6 h H_2_S ventilation group, cytokine counts tended to be lower (Fig 3A). Mechanical ventilation with air induced a vast influx of neutrophils into the bronchoalveolar space compared to controls and all other groups (Fig 3B). Pretreatment with H_2_S for 1, 3, or 5 h reduced neutrophil sequestration, while 6 h ventilation with H_2_S alone nearly prevented neutrophil accumulation (Fig 3B). Compared to controls, air ventilation for 6 h increased ROS production which was reduced by H_2_S pretreatment and 6 h H_2_S ventilation (Fig 3C and 3D).

### Effect of H_2_S posttreatment on physiological parameters

Compared to 6 h air ventilation, 3 h air + 3 h H_2_S ventilation or 6 h H_2_S ventilation alone resulted in reduced Pa_O2_ levels at the end of the experiment (S2 Table). Pa_CO2_ did not differ between groups. pH was only decreased in the 3 h air + 3 h H_2_S and in the 1 h air + 5 h H_2_S groups compared to 6 h air ventilation. MAP was reduced in the 3 h air + 3 h H_2_S and in the 6 h H_2_S groups compared to 6 h air ventilation and in the case of 6 h H_2_S treatment also in comparison to the 5 h air + 1 h H_2_S group. Peak and plateau pressure as well as static compliance did not differ between groups (S2 Table).

### Effect of H_2_S posttreatment on ventilator-induced lung injury

**Fig 4:**
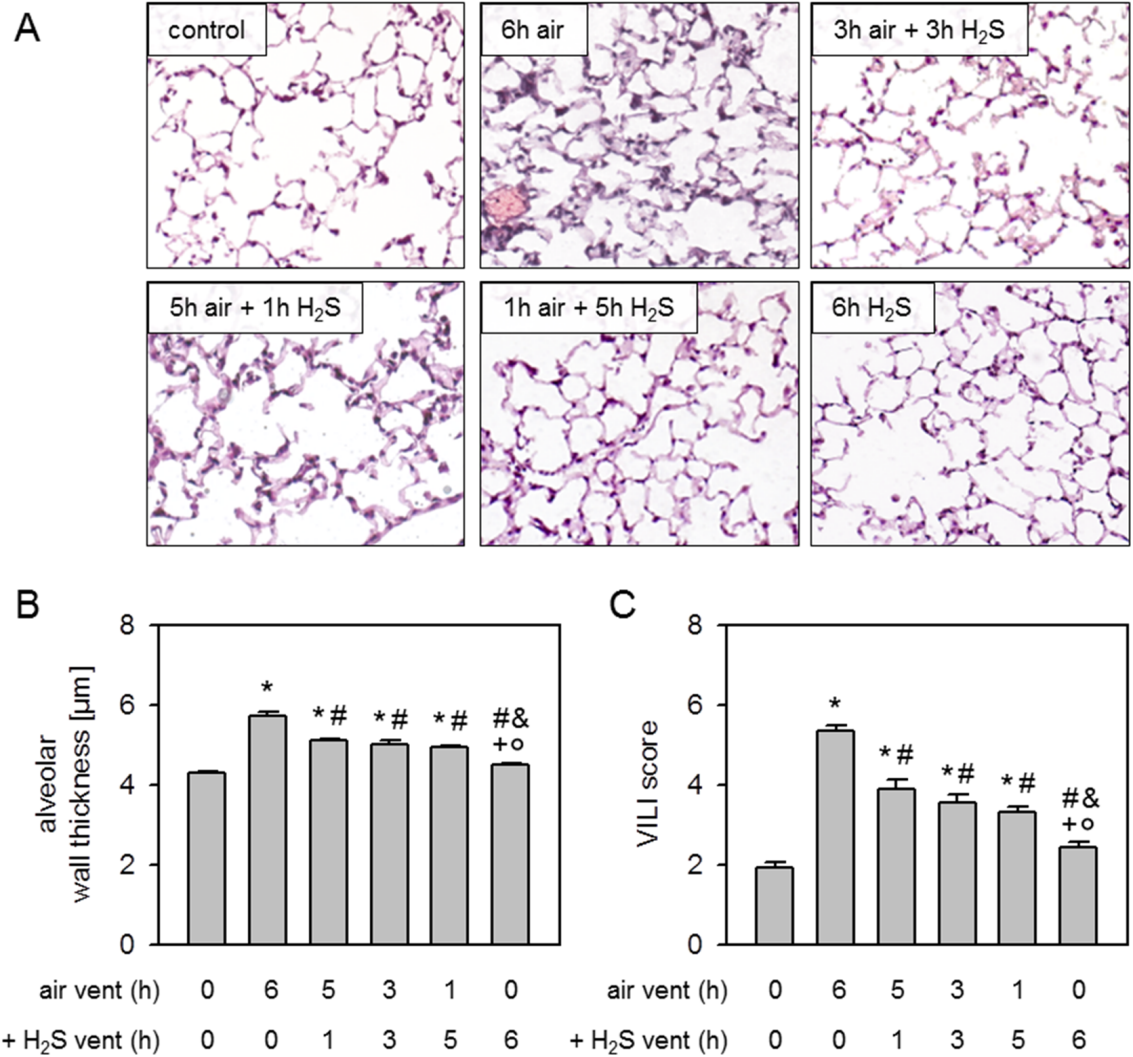
Effect of H_2_S posttreatment on ventilator-induced lung injury. Mice spontaneously breathed air (control) or for 6 h, or they were mechanically ventilated with 12 ml/kg for 6 h either with air alone (6 h air) or air supplemented with 80 ppm H_2_S (6 h H_2_S). All other mice were first mechanically ventilated with air alone for 5, 3, or 1 h, followed by ventilation with 80 ppm H_2_S for another 1, 3, or 5 h as indicated. Lung sections were stained with H&E. Representative pictures are shown for each experimental group as indicated (A). Alveolar wall thickness was measured (B) and ventilator-induced lung injury (VILI) score was calculated (C). Data represent means ± SEM for n = 7/group. ANOVA (Tukey`s post hoc test), *P<0.05 vs. control group; ^#^*P*<0.05 vs. 6h air vent group; ^&^*P*<0.05 vs. 5h air vent + 1h H_2_S vent group; ^+^*P*<0.05 vs. 3h air vent + 3h H_2_S vent group; °*P*<0.05 vs. 1h air vent + 5h H_2_S vent group.

Compared to non-ventilated controls, alveolar wall thickness was substantially increased after 6 h air ventilation. Ventilation with H_2_S for 1, 3, or 5 h after 5, 3, or 1 h air ventilation significantly decreased and 6 h H_2_S ventilation completely prevented alveolar wall thickening (Fig 4Aand 4B). Likewise, there was an increase of the overall VILI score after 6 h air ventilation compared to controls that was reduced by postponed treatment with H_2_S or even prevented by 6 h H_2_S ventilation (Fig 4C).

### Effect of H_2_S posttreatment on lung inflammation and oxidative stress

**Fig 5:**
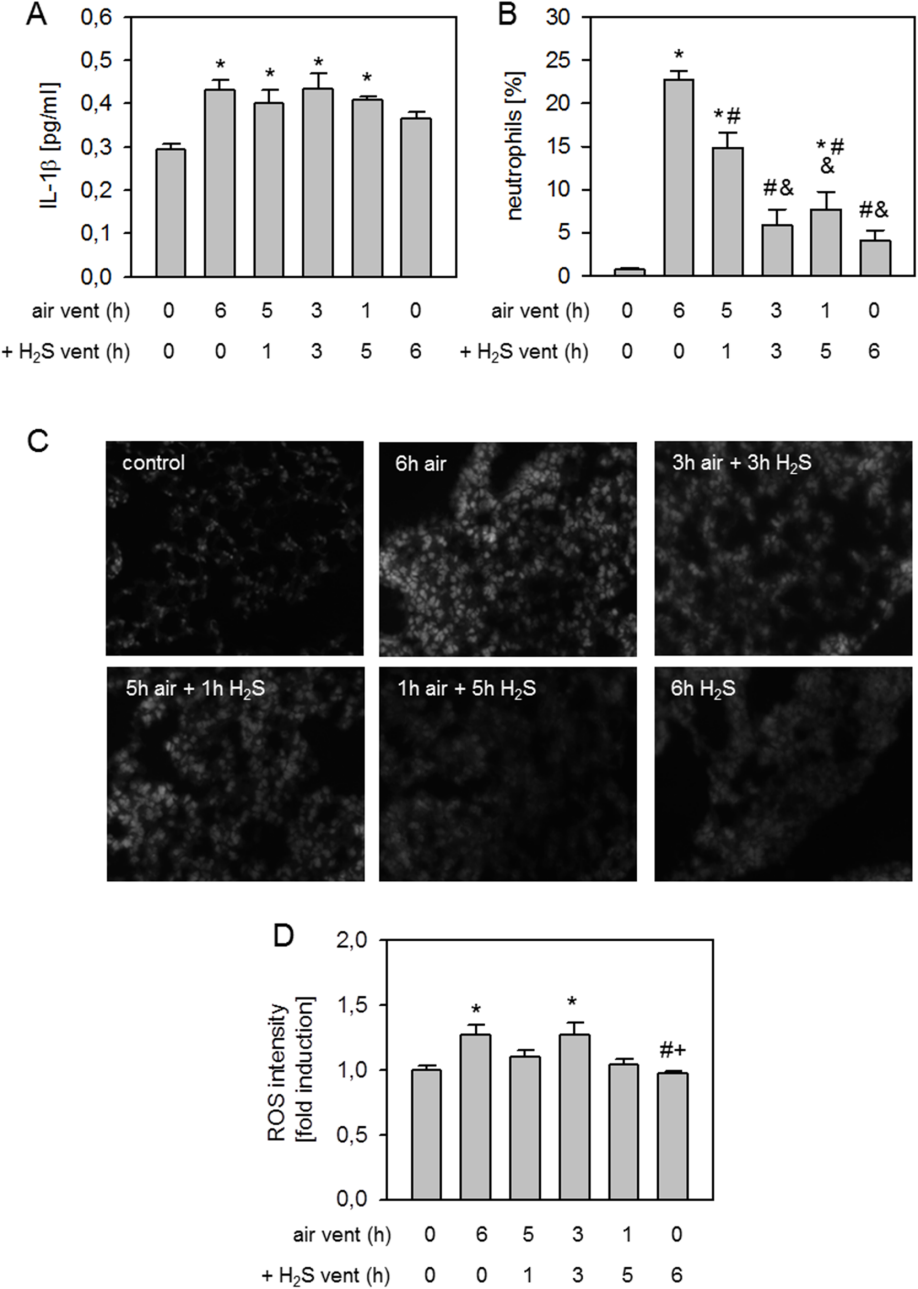
Effect of H_2_S posttreatment on lung inflammation and oxidative stress. Mice spontaneously breathed air (control) or for 6 h, or they were mechanically ventilated with 12 ml/kg for 6 h either with air alone (6 h air) or air supplemented with 80 ppm H_2_S (6 h H_2_S). All other mice were first mechanically ventilated with air alone for 5, 3, or 1 h, followed by ventilation with 80 ppm H_2_S for another 1, 3, or 5 h as indicated. Bronchoalveolar lavage (BAL) IL-1β cytokine content was determined by ELISA (A). The fraction of neutrophil cells was measured in BAL fluid by cytospin analysis (B). Lung tissue sections were stained with dihydroethidium (C). Representative pictures are shown for each experimental group as indicated in C. ROS fluorescence intensity was measured, calculated, and expressed as fold induction compared to control group (D). Data represent means ± SEM for n = 7/group. ANOVA (Tukey`s post hoc test), *P<0.05 vs. control group; ^#^*P*<0.05 vs. 6h air vent group; ^&^*P*<0.05 vs. 5h air vent + 1h H_2_S vent group; ^+^*P*<0.05 vs. 3h air vent + 3h H_2_S vent group.

IL-1β release was induced in all air ventilation groups, irrespective of additional delayed H_2_S treatment compared to non-ventilated controls, while there was a slight reduction in cytokine readings after 6 h of H_2_S ventilation alone (Fig 5A). Neutrophil sequestration was clearly induced by 6 h air ventilation compared to controls. H_2_S ventilation for 1 h after 5 h air ventilation tended to decrease neutrophil accumulation, but did not show statistical significance. When H_2_S inhalation began after 3 or 1 h, or simultaneously with mechanical ventilation, neutrophil influx into the alveolar space was substantially inhibited (Fig 5B).

Compared to controls, formation of reactive oxygen species was augmented in lung tissue after 6 h air ventilation. 5 h air ventilation followed by another 1 h of H_2_S ventilation slightly reduced ROS levels, while 3 h air vent + 3 h H_2_S vent again increased ROS production. 1 h air ventilation followed by 5 h H_2_S ventilation or 6 h H_2_S ventilation completely abolished ROS production, thus ROS detection was comparable to controls (Fig 5C to 5D).

### Effect of expanded H_2_S posttreatment on physiological parameters

In order to answer the question of whether or not the above observed effects of H_2_S posttreatment would be limited to a total experimental time of 6h, two groups were ventilated over 10 h with one group starting H_2_S after 5 h of air ventilation. Compared to 6 h air ventilation, 6 h H_2_S ventilation lowered Pa_O2_ levels (S3 Table), Pa_CO2_ were indifferent between groups. pH was reduced in both 10 h air and 5 h air + 5 h H_2_S groups with respect to 6 h air ventilation. Mean arterial pressure, peak and plateau pressure, and static compliance were not different between groups (S3 Table).

### Effect of expanded H_2_S posttreatment on ventilator-induced lung injury

**Fig 6:**
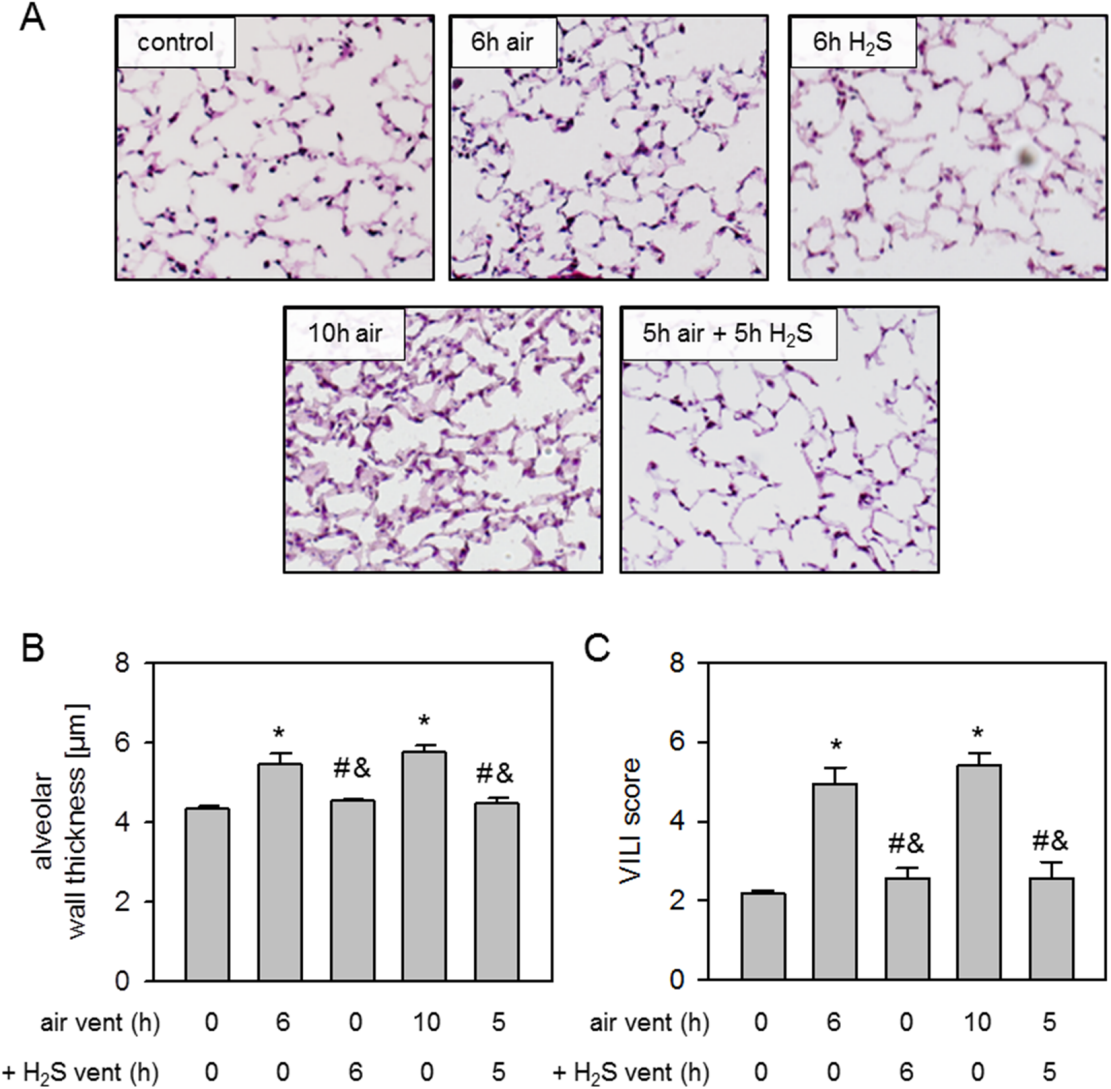
Effect of expanded H_2_S posttreatment on ventilator-induced lung damage. Mice spontaneously breathed air (control) or for 6 h, or they were mechanically ventilated with 12 ml/kg either with air alone (6 h air, 10 h air) or air supplemented with 80 ppm H_2_S (6 h H_2_S). Another group of mice was first mechanically ventilated with air alone for 5 h, followed by ventilation with 80 ppm H_2_S for another 5 h. Lung sections were stained with H&E. Representative pictures are shown for each experimental group as indicated (A). Alveolar wall thickness was measured (B) and ventilator-induced lung injury (VILI) score was calculated (C). Data represent means ± SEM for n = 6/group. ANOVA (Tukey`s post hoc test), *P<0.05 vs. control group; ^#^*P*<0.05 vs. 6h air vent group; ^&^*P*<0.05 vs. 10h air vent group.

Mechanical ventilation for 6 or 10 h with air increased alveolar wall thickness compared to non-ventilated controls which was abolished by 6 h H_2_S and 5 h air ventilation followed by another 5 h of H_2_S ventilation (Fig 6A and 6B). The calculation of an overall VILI score resulted in significantly elevated scores for the 6 and 10 h air ventilation group compared to all other groups (Fig 6C).

### Effect of expanded H_2_S posttreatment on lung inflammation and oxidative stress

**Fig 7:**
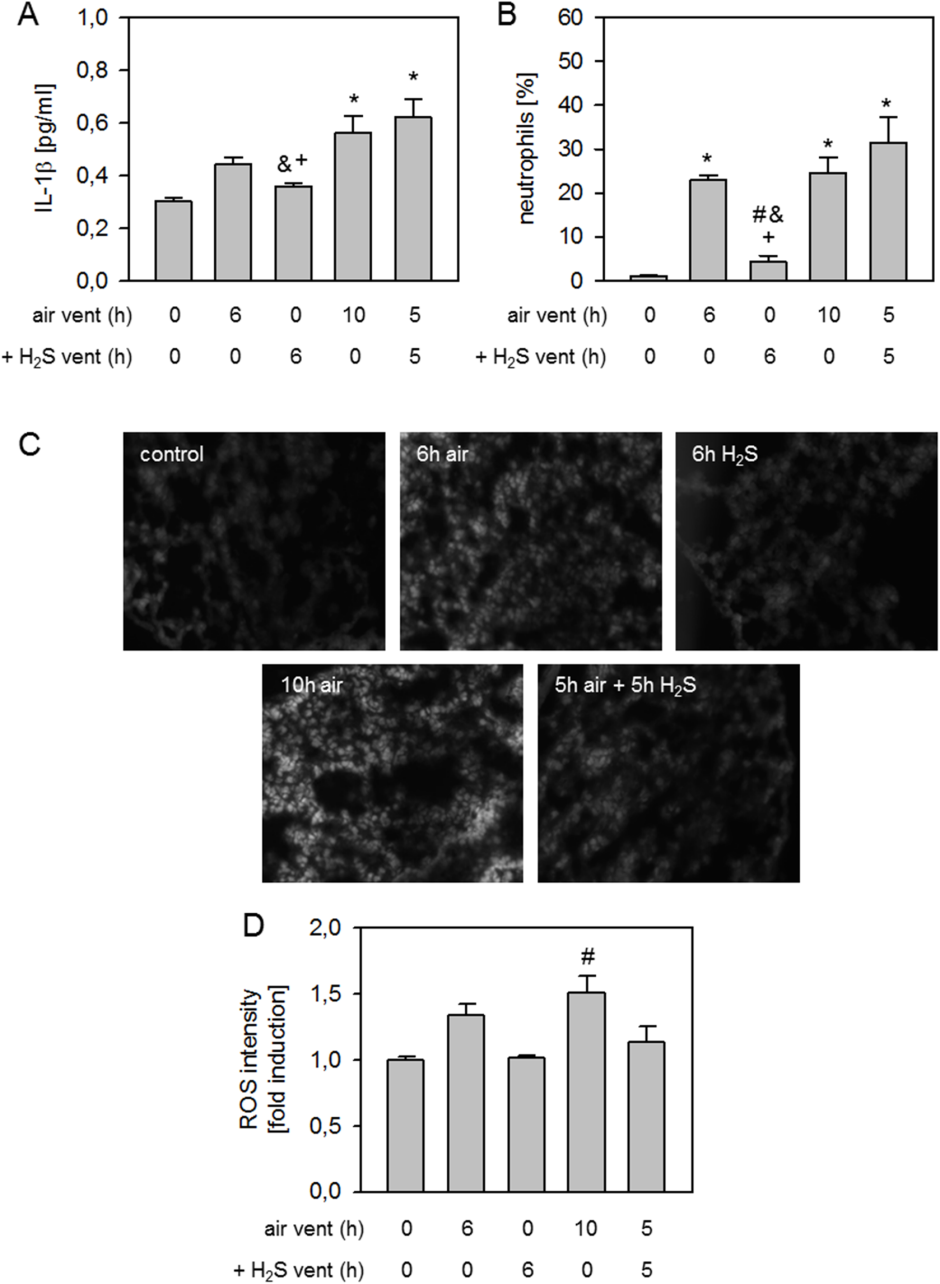
Effect of expanded H_2_S posttreatment on lung inflammation and oxidative stress. Mice spontaneously breathed air (control) or for 6 h, or they were mechanically ventilated with 12 ml/kg either with air alone (6 h air, 10 h air) or air supplemented with 80 ppm H_2_S (6 h H_2_S). Another group of mice was first mechanically ventilated with air alone for 5 h, followed by ventilation with 80 ppm H_2_S for another 5 h. Bronchoalveolar lavage (BAL) IL-1β cytokine content was determined by ELISA (A). The fraction of neutrophil cells was measured in BAL fluid by cytospin analysis (B). Lung tissue sections were stained with dihydroethidium (C). Representative pictures are shown for each experimental group as indicated in C. ROS fluorescence intensity was measured, calculated, and expressed as fold induction compared to control group (D). Data represent means ± SEM for n = 6/group. ANOVA (Tukey`s post hoc test), *P<0.05 vs. control group; ^#^*P*<0.05 vs. 6h air vent group; ^&^*P*<0.05 vs. 10h air vent group; ^+^*P*<0.05 vs. 5h air vent + 5h H_2_S vent group.

Compared to non-ventilated controls, 6 h air ventilation led to a slight increase in IL-1β release, that was again reduced by 6 h H_2_S. Both 10 h air ventilation and 5 h air followed by 5 h H_2_S ventilation further increased IL-1β cytokine levels (Fig 7A). Likewise, neutrophil influx into the bronchoalveolar space was induced by 6h air ventilation compared to non-ventilated controls. 6 h H_2_S ventilation reduced neutrophil sequestration back to control levels. 10 h air ventilation again led to increased neutrophil numbers in the BAL, and the same finding was made when mice received 5 h air ventilation followed by another 5 h of H_2_S ventilation (Fig 7B).

Formation of reactive oxygen species were elevated after 6 h air ventilation compared to non-ventilated controls. 6 h H_2_S ventilation inhibited ROS production. Again, there was a further increase in ROS after 10 h air ventilation as compared to 6 h ventilation and a reduction in ROS levels was observed when mice were ventilated for 5 h with air, followed by 5 h H_2_S ventilation (Fig 7C). These findings were confirmed by densitometric analysis of ROS fluorescence intensity for all groups (Fig 7D).

## Discussion

We have previously shown that inhalation of 80 ppm H_2_S prevents VILI when given during the entire course of mechanical ventilation [11,16,17]. In contrast to higher H_2_S concentrations, no acute or chronic effects were observed by us and others in mice with 80 ppm H_2_S [11,27,28], while lower concentrations failed to mediate lung protection in VILI [14]. However, nothing is known so far about the time-dependent effects of H_2_S treatment in VILI. To answer the question whether pre- or posttreatment as well as shorter application times of H_2_S would have similar effects on VILI, inflammation and oxidative stress, we extensively examined the effects of H_2_S pre- and posttreatment on lung injury, inflammation and oxidation.

### H_2_S pretreatment

Our results suggest that inhalative H_2_S pretreatment protects the lung against VILI. This finding is of increasing interest, since it may support existing or even reveal novel strategies, aiming to diminish forthcoming ventilator-induced lung injury. In the current study, this notion is supported by the observed substantial reduction in edema formation and VILI score compared to non-pretreated mice. These findings are in line with results from animal models of myocardial infarct injury [29] and hepatic [24], cardiac [30], cerebral [25], or retinal ischemia-reperfusion injury [20]. Here, H_2_S preconditioning clearly prevented organ injury. Our results further suggest that the longer H_2_S is applied prior to the onset of ventilation, the more protection is achieved. This observation appears interesting, because gaseous H_2_S is rapidly eliminated and extending the time of H_2_S inhalation should not result in accumulation of the molecule in mammalian cells [9]. A possible explanation for the observed time-dependency could be seen in differential gene expression patterns upon H_2_S application, in that upregulation or suppression of H_2_S sensitive genes might count for increased lung protection. This notion is supported by our previously published data on H_2_S mediated gene expression [16] and by a recent study by Roberts et al. [31]. In the latter report, rats inhaled 200 ppm H_2_S for 3 h per day on up to 5 consecutive days and gene expression patterns in the nasal respiratory epithelium shifted over time from genes involved in inflammation and anti-oxidation to genes associated with matrix remodeling.

It is well known that cyclic stretching of the lung leads to an inflammatory response [5,6]. We have previously shown that H_2_S inhalation inhibits pro-inflammatory cytokine release and neutrophil transmigration during mechanical ventilation [11,17]. Likewise, in the current study we observed increased IL-1β and neutrophils in the BAL which were decreased in the presence of 6 h H_2_S inhalation. Thus, continuous H_2_S application appears to inhibit the inflammatory response upon mechanical ventilation. Interestingly, H_2_S pretreatment did not affect IL-1β release. These findings are in contrast to previously reported data. In a cerebral ischemia-reperfusion injury model, pretreatment with 40 ppm H_2_S for 8 h each day for 7 days reduced IL-6 and TNF-α cytokine release [25]. Similar results were found in a rat model of small intestine ischemia-reperfusion injury [21]. There, rats were treated with the soluble H_2_S donor sodium hydrosulfide 24 h prior to ischemia-reperfusion and TNF-α release was diminished [21]. In the light of these findings, we suppose that IL-1β might either not be directly involved in the protective effects observed, or measuring IL-1β at the end of the experiment was too late to detect important mechanistic differences between groups. Another key step in the development of VILI and inflammation is reflected by neutrophil transmigration into the bronchoalveolar space [5]. With respect to H_2_S preconditioning, neutrophil numbers have not been examined in other studies. However, in models of small intestine ischemia-reperfusion injury, sodium hydrosulfide application 24 h prior to ischemia-reperfusion clearly inhibited leukocyte rolling and adhesion [22,23]. In the current study, H_2_S pretreatment clearly reduced neutrophil infiltration, irrespective of the duration of pretreatment, strongly suggesting that H_2_S inhalation prior to the onset of ventilation is sufficient to inhibit inflammatory responses during mechanical ventilation.

The excessive production of ROS due to cyclic stretching is clearly involved in the exacerbation of inflammation and VILI [7]. Here we demonstrate that H_2_S pretreatment prevents the increase of ROS formation. Recent findings from our lab point in the same direction: H_2_S treatment inhibited excessive ROS production in both ventilator- [17] and hyperoxia-induced lung injury [13] which was associated with lung protection. These findings underline the highly anti-oxidative capability of exogenous H_2_S *in vivo*. A few previously published studies using other models, other species and *in vitro* experiments underscore our results. For instance, Ji *et al*. observed in a rat model of cerebral ischemia-reperfusion injury that malondialdehyd and 8-hydroxy-2'-deoxyguanosine readings, as markers for oxidative stress, were reduced due to H_2_S inhalation for 8 h per day on 7 days prior to ischemia-reperfusion [25]. Incubation of neuronal cells [32–34], H9c2 cardiomyoblasts [35,36], or kidney epithelial cells [37] with H_2_S prior to injurious treatment resulted in a marked decrease of ROS production, which was accompanied by cell protection. Here, we extended our recent findings and show for the first time even that H_2_S preconditioning is sufficient to reduce ROS formation in VILI, suggesting that this step might also be involved in reducing the inflammatory response and mediating lung protection.

### H_2_S Posttreatment

In order to answer the question whether or not H_2_S has the potential to limit lung injury when given after the insult has been set, animals were posttreated with H_2_S after the onset of mechanical ventilation. In the present study, we were able to demonstrate that even postponing H_2_S application reduces lung injury in a time-dependent fashion, underlining the effective and therapeutic potential of H_2_S. In a long term model of myocardial infarction, application of 3 boluses of sodium hydrosulfide on 3 consecutive days after the initial trauma was also sufficient to reduce infarct size [29]. However, our findings are in contrast to a more recent study that revealed no improvement of histological signs of lung injury when soluble sulfide was administered continuously, starting directly after induction of blunt chest trauma [38]. To the best of our knowledge, no data exist for the effect of H_2_S posttreatment in VILI. In the present study, we clearly show that H_2_S posttreatment protects against VILI.

It is likely that regulation of the inflammatory response has a significant role in the orchestration of posttreatment organ protection by H_2_S [9]. However, and as is our observation in H_2_S pretreatment, H_2_S showed only a reducing effect on IL-1β release when given with the onset of ventilation and continued over the entire ventilation period. Postponing H_2_S administration did not affect the cytokine release in our model. The above mentioned study on posttreatment in blunt chest trauma demonstrated similarly inconsistent results for lung cytokine release. In plasma, IL-6 was unaffected by H_2_S, while the anti-inflammatory cytokine IL-10 was clearly elevated, suggesting at least a systemic anti-inflammatory role for H_2_S posttreatment in lung trauma [38]. In our study, neutrophil transmigration into the alveolar space was diminished by H_2_S posttreatment. The longer and earlier H_2_S was applied for, the more pronounced was the effect. Because neutrophils were found to start transmigration into the bronchoalveolar space as early as 4 h after the onset of mechanical ventilation in our model, it is not surprising that H_2_S posttreatment failed to completely prevent neutrophil sequestration when given in the final hour of ventilation. The marked decrease in neutrophil numbers compared to ventilation alone may be explained by a direct impact of H_2_S on leukocyte transmigration capacity, as described for H_2_S pretreatment [23,39].

As discussed above, the oxidative responses are strongly associated with inflammation and the development of lung injury upon mechanical ventilation. The role of H_2_S posttreatment in regulating excessive ROS production remains unclear. In a rat model of lung injury induced by blast limb trauma, oxidative stress was characterized by malondialdehyd content and superoxide dismutase activity. Here, H_2_S posttreatment reduced oxidative stress as well as lung injury [40]. Our data point in the same direction and clearly show that even postponed H_2_S treatment is able to inhibit ROS production and oxidative stress. Somewhat surprising is the observation that starting H_2_S application in the middle of the ventilation period, i.e., at 3 h, did not limit ROS production in a linear time-response function. The reason for this result may be based on experimental variability but remains speculative. Nonetheless, in our setting 1 h of treatment at the end of ventilation was sufficient to reduce ROS formation, underlining the potential of H_2_S to interfere with the production of free radicals. One possible mechanism may be H_2_S-induced upregulation of Akt phosphorylation [17].

Taking the above discussed results into account, we finally hypothesized that (1) H_2_S might even affect VILI when applied at a time point at which lung injury has already been fully established and (2) this effect would be even more pronounced, if H_2_S were to be continuously given for several hours. Therefore, we prolonged the total ventilation time in a third set of experiments to 10 h and began to apply H_2_S as late as after 5 h. The data show clearly that even postponing H_2_S inhalation for 5 h during injurious ventilation finally leads to substantial protection against lung damage. Most interestingly, the observed protection appears independent of the anti-inflammatory effects of H_2_S, because neither cytokine release nor neutrophil accumulation was affected by H_2_S as compared to ventilation alone. However, ROS formation was completely prevented supporting the hypothesis that oxidative stress is the key mediator in VILI and that the anti-oxidative action of H_2_S represents the major protective mechanism.

## Conclusions

The study examines the protective effects of inhaled H_2_S in VILI in both a pre- as well as posttreatment setting. We show for the first time that pretreatment with H_2_S time-dependently prevents lung damage by inhibiting inflammation and ROS formation. The same is true when H_2_S administration starts after the onset of injury, even when given at a time point when lung injury has already occurred. The inhibition of oxidative stress by H_2_S appears to account for H_2_S mediated lung protection. Taken together, H_2_S pre- or posttreatment displayed therapeutic potential against lung injury under experimental conditions.

## Supporting information

S1 Table Effect of H_2_S pretreatment on physiological parameters. Mice were mechanically ventilated with 12ml/kg for 6h either with air alone (6h air) or air supplemented with 80ppm H_2_S (6h H_2_S). All other mice spontaneously breathed air supplemented with 80ppm H_2_S 1h, 3h, or 5h prior to mechanical ventilation with air for another 6h as indicated. pH, arterial oxygen partial pressure (Pa_O2_), and arterial carbon dioxide partial pressure (Pa_CO2_) were measured at the end of the experiment. Peak pressure (P_Peak_), plateau pressure (P_Plateau_), mean arterial pressure (MAP), and static compliance (C_stat_) were monitored throughout ventilation and depicted as 6h average. Data represent means ± SEM for n = 5-7/group. ANOVA (Tukey`s post hoc test), ^#^*P*<0.05 vs. 6h air vent group.
(TIF)

S2 Table Effect of H_2_S posttreatment on physiological parameters. Mice were mechanically ventilated with 12ml/kg for 6h either with air alone (6h air) or air supplemented with 80ppm H_2_S (6h H_2_S). All other mice were first mechanically ventilated with air alone for 5, 3, or 1h, followed by ventilation with 80ppm H_2_S for another 1, 3, or 5h as indicated. pH, arterial oxygen partial pressure (Pa_O2_), and arterial carbon dioxide partial pressure (Pa_CO2_) were measured at the end of the experiment. Peak pressure (P_Peak_), plateau pressure (P_Plateau_), mean arterial pressure (MAP), and static compliance (C_stat_) were monitored throughout ventilation and depicted as 6h average. Data represent means ± SEM for n = 4-7/group. ANOVA (Tukey`s post hoc test), ^#^*P*<0.05 vs. 6h air group; ^&^*P*<0.05 vs. 5h air + 1h H_2_S group.
(TIF)

S3 Table Effect of expanded delayed H_2_S treatment on physiological parameters. Mice were mechanically ventilated with 12ml/kg either with air alone (6h air, 10h air) or air supplemented with 80ppm H_2_S (6h H_2_S). Another group of mice was first mechanically ventilated with air alone for 5h, followed by ventilation with 80ppm H_2_S for another 5h. pH, arterial oxygen partial pressure (Pa_O2_), and arterial carbon dioxide partial pressure (Pa_CO2_) were measured at the end of the experiment. Peak pressure (P_Peak_), plateau pressure (P_Plateau_), mean arterial pressure (MAP), and static compliance (C_stat_) were monitored throughout ventilation and depicted as 6h average. Data represent means ± SEM for n = 4-6/group. ANOVA (Tukey`s post hoc test), ^#^*P*<0.05 vs. 6h air group.
(TIF)
