# Correction: Pre- and posttreatment with hydrogen sulfide prevents ventilator-induced lung injury by limiting inflammation and oxidation

**DOI:** 10.1371/journal.pone.0186532

**Published:** 2017-10-11

**Authors:** Simone Faller, Raphael Seiler, Rosa Donus, Helen Engelstaedter, Alexander Hoetzel, Sashko Gregoriev Spassov

There is an error in the authors’ affiliation. The correct affiliation is: Department of Anesthesiology and Critical Care, Medical Center–University of Freiburg, Faculty of Medicine, University of Freiburg, Germany.
