# Supplementary figures and images for: Pre- and posttreatment with hydrogen sulfide prevents ventilator-induced lung injury by limiting inflammation and oxidation
Source: PLoS One. 2017 Apr 28;12(4):e0176649. doi: 10.1371/journal.pone.0176649 (PMC5409137; doi:10.1371/journal.pone.0176649)

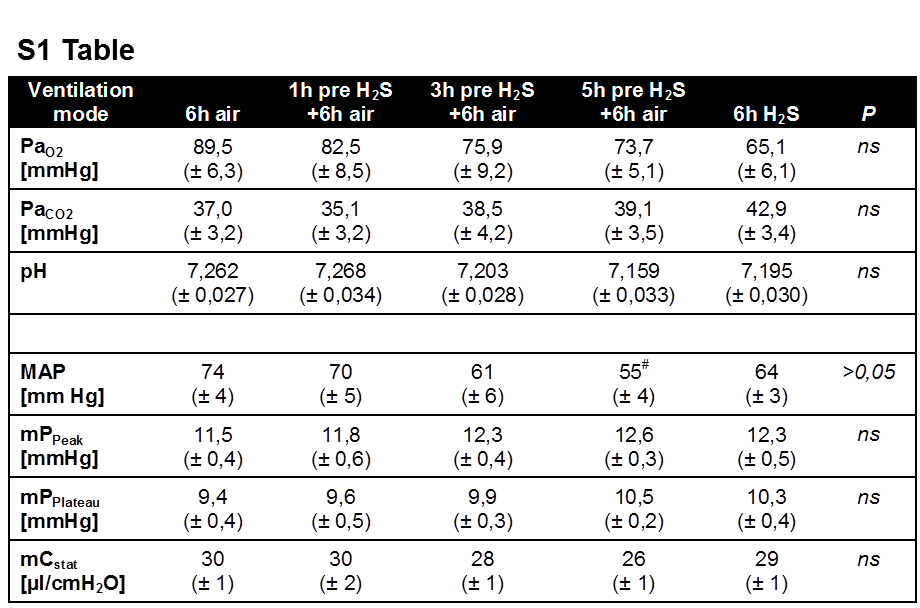

Supplement: S1 Table — Mice were mechanically ventilated with 12ml/kg for 6h either with air alone (6h air) or air supplemented with 80ppm H2S (6h H2S). All other mice spontaneously breathed air supplemented with 80ppm H2S 1h, 3h, or 5h prior to mechanical ventilation with air for another 6h as indicated. pH, arterial oxygen partial pressure (PaO2), and arterial carbon dioxide partial pressure (PaCO2) were measured at the end of the experiment. Peak pressure (PPeak), plateau pressure (PPlateau), mean arterial pressure (MAP), and static compliance (Cstat) were monitored throughout ventilation and depicted as 6h average. Data represent means ± SEM for n = 5-7/group. ANOVA (Tukey`s post hoc test), #P<0.05 vs. 6h air vent group. (TIF) [file pone.0176649.s001.tif]

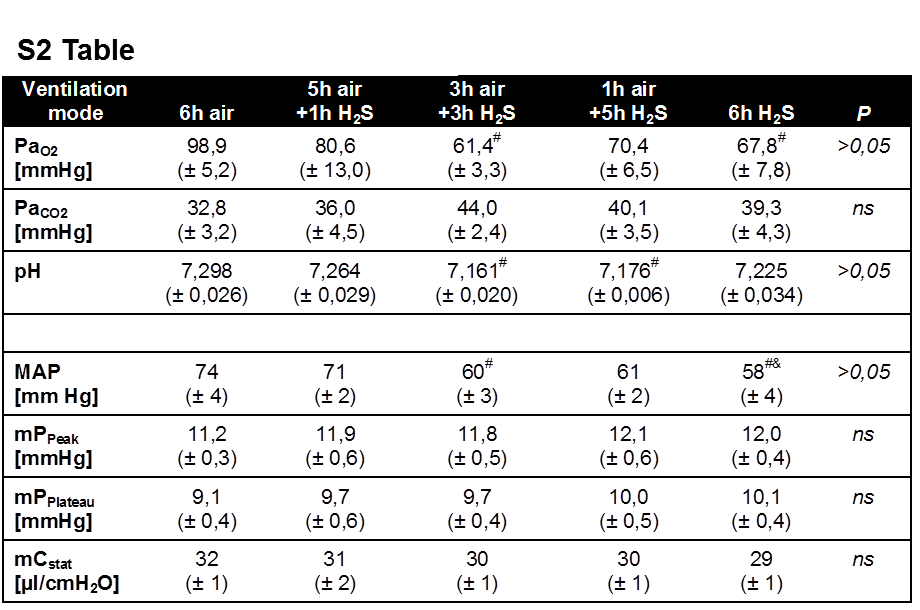

Supplement: S2 Table — Mice were mechanically ventilated with 12ml/kg for 6h either with air alone (6h air) or air supplemented with 80ppm H2S (6h H2S). All other mice were first mechanically ventilated with air alone for 5, 3, or 1h, followed by ventilation with 80ppm H2S for another 1, 3, or 5h as indicated. pH, arterial oxygen partial pressure (PaO2), and arterial carbon dioxide partial pressure (PaCO2) were measured at the end of the experiment. Peak pressure (PPeak), plateau pressure (PPlateau), mean arterial pressure (MAP), and static compliance (Cstat) were monitored throughout ventilation and depicted as 6h average. Data represent means ± SEM for n = 4-7/group. ANOVA (Tukey`s post hoc test), #P<0.05 vs. 6h air group; &P<0.05 vs. 5h air + 1h H2S group. (TIF) [file pone.0176649.s002.tif]

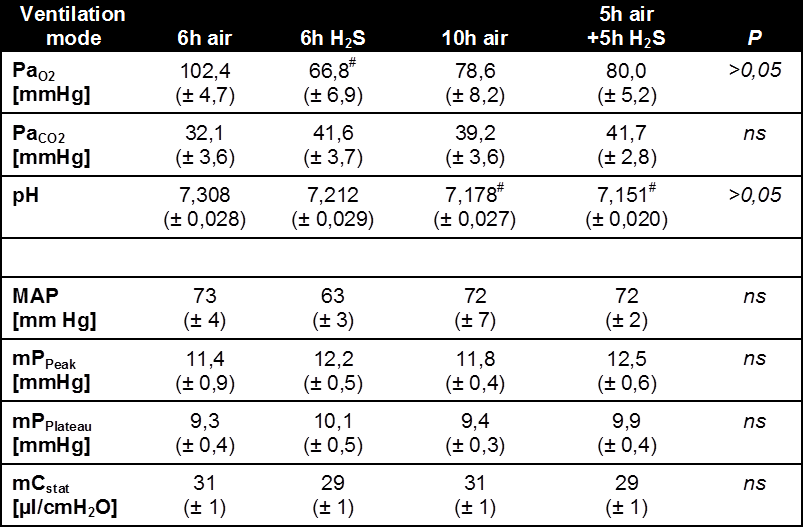

Supplement: S3 Table — Mice were mechanically ventilated with 12ml/kg either with air alone (6h air, 10h air) or air supplemented with 80ppm H2S (6h H2S). Another group of mice was first mechanically ventilated with air alone for 5h, followed by ventilation with 80ppm H2S for another 5h. pH, arterial oxygen partial pressure (PaO2), and arterial carbon dioxide partial pressure (PaCO2) were measured at the end of the experiment. Peak pressure (PPeak), plateau pressure (PPlateau), mean arterial pressure (MAP), and static compliance (Cstat) were monitored throughout ventilation and depicted as 6h average. Data represent means ± SEM for n = 4-6/group. ANOVA (Tukey`s post hoc test), #P<0.05 vs. 6h air group. (TIF) [file pone.0176649.s003.tif]
